# Patent Ductus Arteriosus with Left to Right Shunting Exacerbating Aortic Valve Stenosis

**DOI:** 10.14797/mdcvj.1514

**Published:** 2025-05-01

**Authors:** Paulamy Ganguly, Zhihao Zhu, Suhas Babu, Nandan Shettigar, Valeria Duarte, C. Huie Lin

**Affiliations:** 1 Texas A&M School of Engineering Medicine, Houston, Texas, US; 2 Methodist DeBakey Heart & Vascular Center, Houston Methodist Hospital, Houston, Texas, US

**Keywords:** aortic valve stenosis, patent ductus arteriosus, congenital heart disease, interventional cardiology, PDA closure, TAVR

## Abstract

Aortic stenosis (AS) leads to a reduced effective orifice of the aortic valve, and severity is based on obstructions in flow and velocity. In some patients, coexisting structural cardiac abnormalities that increase left ventricular volume, such as patent ductus arteriosus (PDA), may complicate evaluation and management. We present the case of a patient with severe AS and unrepaired PDA and discuss the hemodynamic implications and important physiological changes resulting from the interactions between these lesions. It is important for clinicians to consider the impact of PDA closure in the evolution of AS and related symptoms.

## Introduction

Senile degenerative aortic stenosis (AS) leads to a reduction of the effective orifice of the aortic valve. This can increase both the transvalvular pressure gradient and the valve jet velocity. The clinical history of AS typically involves a long asymptomatic period, with symptoms such as exertional dyspnea, angina, and exertional presyncope/syncope starting to manifest as the disease becomes more severe.^1^ When AS progresses to hemodynamically significant left ventricle outflow tract (LVOT) obstruction, the increased LV afterload can also result in LV remodeling and dysfunction. Patients with severe symptomatic AS will go on to develop heart failure or even death unless they undergo aortic valve replacement (AVR).^2^,^3^ Another consequence of severe AS is compensatory pulmonary hypertension, which results from increased pulmonary vascular resistance (PVR) and subsequent right ventricle dysfunction with more advanced disease.^4^

In some patients, coexistence of other hemodynamically significant structural cardiac abnormalities, such as patent ductus arteriosus (PDA), may alter the physiology of AS and complicate evaluation and management. In PDA, for example, the fetal blood vessel between the aorta and pulmonary artery that usually closes spontaneously after birth remains open. One of the most common congenital heart deformities, PDA has an incidence of 1:2000, making up 5% to 10% of all congenital heart diseases.^5^,^6^ The clinical manifestations of PDA are primarily related to the degree of post-tricuspid left-to-right shunting, typically inducing left-sided volume overload when significant. Patients can range from asymptomatic, with incidental small shunts (Qp/Qs < 1.5), to moderate (Qp/Qs between 1.5 and 2.2) and large shunts (Qp/Qs > 2.2) that may present with clinical heart failure, left-sided volume overload, and pulmonary arterial hypertension.^7^ We present the case of a patient with severe AS and PDA and the complex interplay of the pathophysiology of these two lesions.

## Case Presentation

### History of presentation

**Figure 1:**
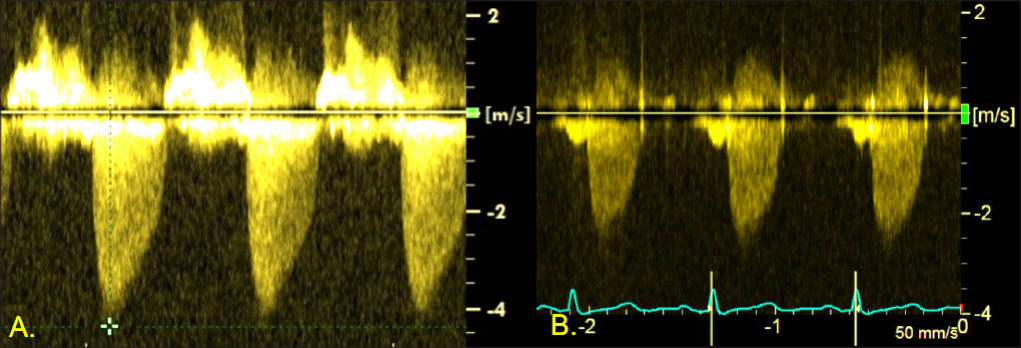
Doppler echocardiography of stenotic aortic valve. **(A)** Baseline severe aortic stenosis. **(B)** Follow-up post-procedure echocardiography.

A 74-year-old woman with severe asymptomatic AS was referred for evaluation and incidentally identified PDA. Her past medical history was significant for rheumatoid arthritis, hyperlipidemia, and AS. Previous serial transthoracic echocardiography had demonstrated progressive aortic valve stenosis first noted as mild 3 years ago. However, most recent echocardiography (Figure 1A) findings demonstrated severe AS (peak velocity 4.2 m/s, mean gradient 47 mm Hg, Table 1, Row 1) as well as a PDA with left-to-right shunt, subsequently confirmed on diagnostic catheterization.

**Table 1:** Echocardiography results pre- and post-intervention.


	AORTIC VALVE AREA	AORTIC VALVE MAXIMUM VELOCITY	AORTIC VALVE MEAN PEAK GRADIENT	AORTIC VALVE MAXIMUM PEAK GRADIENT

Baseline	0.7 cm^2^	4.2 m/s	47 mm Hg	81 mm Hg

Post PDA closure	1.0 cm^2^	2.7 m/s	18 mm Hg	30 mm Hg


### Differential Diagnosis

The worsening of AS was attributed to natural history and progression of senile degenerative AS versus the hemodynamic effects of increased flow due to the PDA.

### Investigations

**Figure 2:**
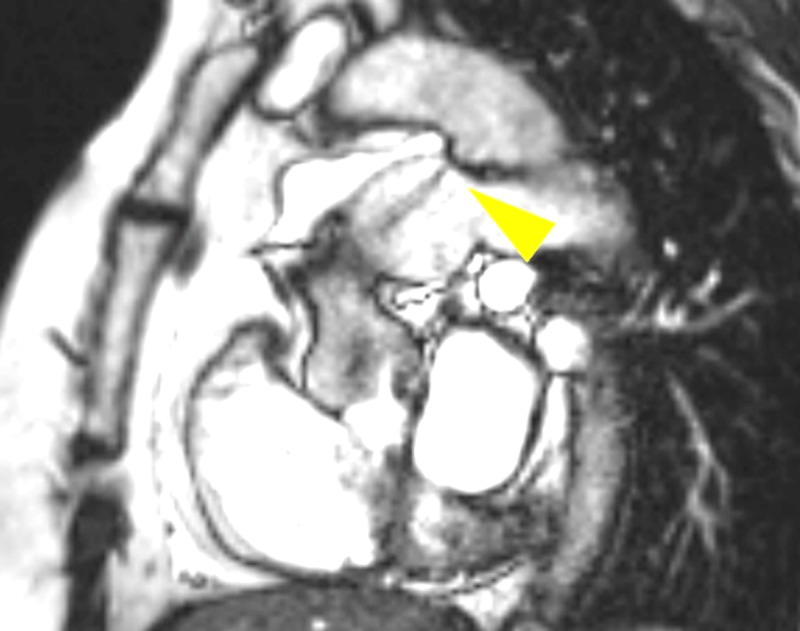
Cardiac magnetic resonance imaging showing patent ductus arteriosus with left-to-right shunting. Yellow triangle indicates flow jet from the aorta to the pulmonary artery.

Cardiac magnetic resonance imaging (Figure 2) showed PDA with left-to-right shunt (Qp/Qs 1.8), mild LA, LV dilation (LV end diastolic volume 107 mL/m^2^) and an ejection fraction of 56%. It was suspected that the degree of left-to-right shunt led to a left-sided volume overload that exacerbated the aortic valve pressure gradient. She was taken to cardiac catheterization lab for hemodynamic evaluation and PDA closure, if indicated.

### Management (Medical/Interventions)

**Figure 3:**
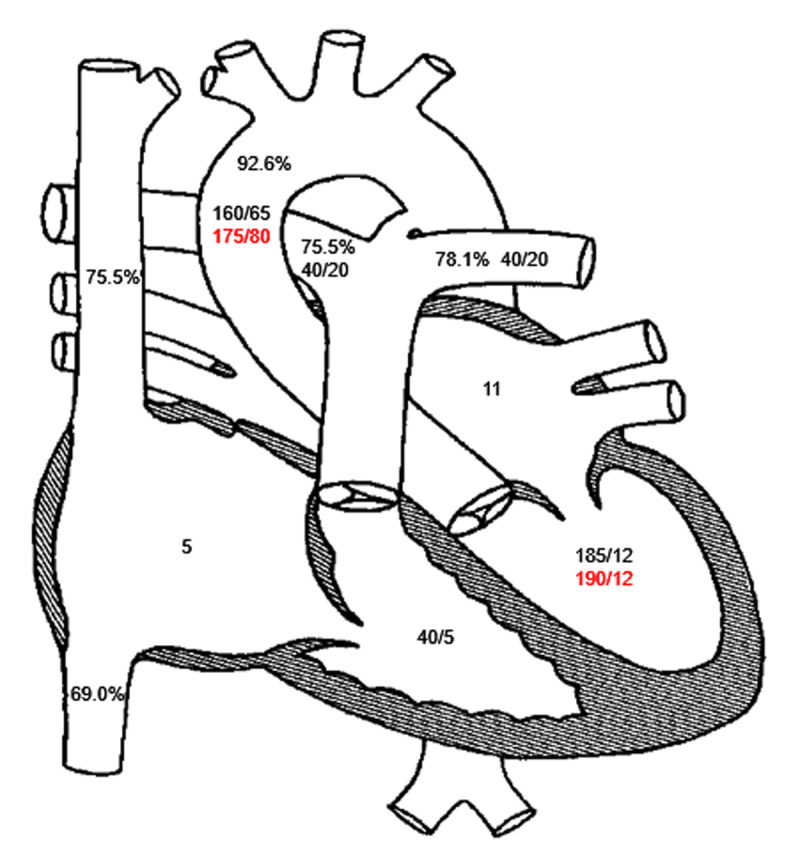
Invasive hemodynamics. oximetry and invasive hemodynamics, with baseline readings in black and post-closure readings in red.

**Figure 4:**
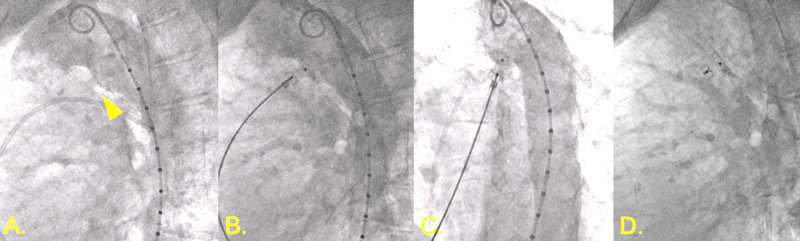
Patent ductus arteriosus (PDA) closure. **(A)** Angiography demonstrating 5.5 mm calcified PDA (triangle). **(B,C)** Amplatzer 10-8 occluder with aortic disc in descending aorta with aortography demonstrating no further shunting. **(D)** Stable position of PDA occludes device following release.

Invasive hemodynamics and oximetry pre- and post-closure (Figure 3) demonstrated improvement of the AS gradient from 25 mm Hg to 15 mm Hg post-intervention. The PDA was successfully crossed and closed using an Amplatzer ductal occluder (Abbott Cardiovascular) (Figure 4).^8^,^9^,^10^ The patient tolerated the procedure without any complications.

## Discussion

We describe the case of a 74-year-old female with severe AS (peak velocity 4.2 m/s) with exacerbation of aortic valve hemodynamics due to left-to-right shunting caused by PDA.^8^ This unique case demonstrates how a PDA with hemodynamically significant shunting caused left-sided volume overload with normal PVR. While this did not cause frank heart failure symptoms, it resulted in worsened aortic valve hemodynamics with elevated pressure gradient across the valve (Table 1). This exacerbation of aortic valve hemodynamics represented a different manifestation from the more commonly seen large PDA, which can cause heart failure symptoms or progression to pulmonary arterial hypertension. In patients with AS complicated by PDA (versus isolated AS), the flow across the aortic valve is influenced by the size, length, stiffness, and shunting of additional blood.^9^ With increased flow to the left circulation from the shunt, there is an increase in volume and, therefore, jet velocity of flow across the aortic valve.^9^ As seen in this patient, after successful occlusion of PDA, there was notable improvement of AS parameters (peak velocity 2.7 m/s) consistent with mild AS (compared to severe AS pre-closure). Without any intervention to the valve itself, occlusion of the shunt reduced left-sided volume overload and subsequently decreased pressure gradient and flow velocity.

Senile AS with coexisting PDA is a rare phenomenon that leads to this unique presentation. Previously reported cases of similar concomitant presentations involved younger patients with bicuspid aortic valve. Anatomically, patients with concurrent PDA and AS may have LV hypertrophy and increased LV ejection fraction compared to patients with PDA alone.^10^

**Figure 5:**
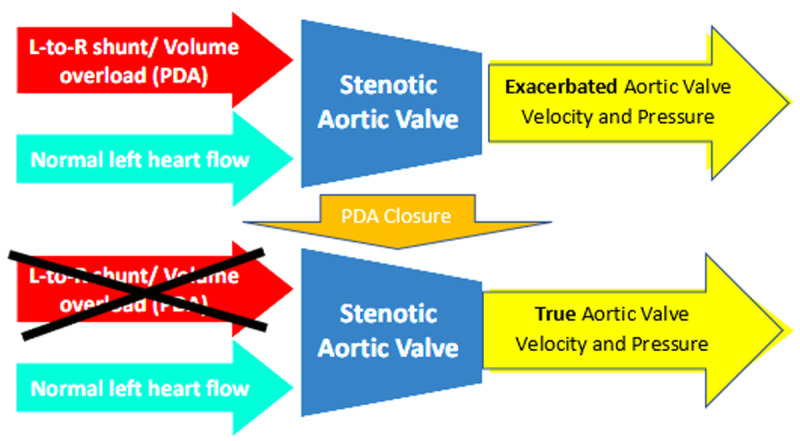
Diagram demonstrating impact of patent ductus arteriosus (PDA) closure. Prior to PDA closure, the left-to-right shunt, resulting in increased volume through the stenotic aortic valve, exacerbated the aortic valve velocity and pressure. Once the PDA is closed, the true aortic valve velocity and pressure are measured.

A key takeaway of this case is the dependence of current determination of AS severity on flow and how this can be perturbed by changes in left-sided volume/flow, such as in the presence of a post-tricuspid shunt like PDA. Systematic hemodynamic evaluation is required to determine whether there is severe intrinsic aortic valve disease that requires AVR versus shunt-related volume load, exacerbating aortic velocity and pressure gradients. Percutaneous closure of the PDA is indicated for significant left-to-right shunts for symptom relief, decreasing the likelihood of progression to PAH or heart failure. In this particular case, it also reduced the impression of the severity of AS, decreasing peak velocity and gradient, all without valve intervention.^11^ While advances in transcatheter AVR have made valve replacements more feasible and accessible for older patients, PDA closure in this patient allowed the appropriate deferral of aortic valve intervention (Figure 5).

## Follow-up

Post-procedure echocardiography (Figure 1B) showed improvement of aortic valve velocity and gradient (peak velocity 2.7 m/s, mean gradient 18 mm Hg, Table 1, Row 2). She was started on aspirin 81 mg daily and ticagrelor 90 mg twice daily. She was switched to ticagrelor after having a rash while on clopidogrel. She was instructed to discontinue ticagrelor 30 days following the procedure. At her 2-month post-procedure follow-up visit, she denied any cardiac symptoms such as dyspnea on exertion, chest pain, palpitations, and dizziness/syncope. Her blood pressure was well controlled with a home regimen of losartan 100 mg daily. She reported good exercise tolerance and could perform activities of daily living, including keeping up with her grandchildren.

## Conclusion

Our case demonstrates how a left-to-right shunt in PDA (or other post-tricuspid shunts) may exacerbate AS hemodynamics. Successful occlusion of the shunt in this case may have avoided early/unnecessary referral for AVR and improved clarity in hemodynamic monitoring of AS progression.
